# An Overview of the Health Effects of Bisphenol A from a One Health Perspective

**DOI:** 10.3390/ani13152439

**Published:** 2023-07-28

**Authors:** Ana M. Molina-López, Francisca Bujalance-Reyes, Nahúm Ayala-Soldado, Rafael Mora-Medina, Antonio Lora-Benítez, Rosario Moyano-Salvago

**Affiliations:** 1 Departamento Anatomía y Anatomía Patológica Comparadas y Toxicología, Unidad de Investigación Competitiva Zoonosis y Enfermedades Emergentes desde la Perspectiva de Una Salud ENZOEM, Universidad de Córdoba, Campus de Rabanales, Edificio Darwin, E-14071 Córdoba, Spain; r.moyano@uco.es; 2 Departamento Anatomía y Anatomía Patológica Comparadas y Toxicología, Universidad de Córdoba, Campus de Rabanales, Edificio Darwin, E-14071 Córdoba, Spain; v02buref@uco.es (F.B.-R.); v02momer@uco.es (R.M.-M.); v12lobea@uco.es (A.L.-B.)

**Keywords:** bisphenol A, One Health, transgenerational, reproductive effects, metabolic disorder

## Abstract

**Simple Summary:**

Endocrine disruptors are substances with a capacity to alter the endocrine system, one of the best known being bisphenol A. Bisphenol A is employed in the manufacture of a multitude of utensils used daily, and it is constantly being found polluting the environment and food. It is also an important aquatic pollutant, and its presence has been detected in coastal areas, rivers, and streams, among others. Due to its ubiquity, human and animal populations are frequently exposed to this compound. This work has compiled the effects on health of this substance, which are highly varied and are relevant in affecting reproduction and causing metabolic or immune system alterations, among others, as well as related to an increase in hormone-dependent pathologies, obesity, or type 2 diabetes. Monitoring the populations at risk and establishing a safe exposure level are proposed as being fundamental points in the control of exposure to this compound.

**Abstract:**

Bisphenol A (BPA) is a chemical compound, considered as an “emerging pollutant”, that appears ubiquitously, contaminating the environment and food. It is an endocrine disruptor, found in a multitude of consumer products, as it is a constituent of polycarbonate used in the manufacture of plastics and epoxy resins. Many studies have evaluated the effects of BPA, using a wide range of doses and animal models. In this work, we carried out a review of relevant research related to the effects of BPA on health, through studies performed at different doses, in different animal models, and in human monitoring studies. Numerous effects of BPA on health have been described; in different animal species, it has been reported that it interferes with fertility in both females and males and causes alterations in their offspring, as well as being associated with an increase in hormone-dependent pathologies. Similarly, exposure to BPA has been related to other diseases of great relevance in public health such as obesity, hypertension, diabetes, or neurodevelopmental disorders. Its ubiquity and nonmonotonic behavior, triggering effects at exposure levels considered “safe”, make it especially relevant when both animal and human populations are constantly and inadvertently exposed to this compound. Its effects at low exposure levels make it essential to establish safe exposure levels, and research into the effects of BPA must continue and be focused from a “One Health” perspective to take into account all the factors that could intervene in the development of a disease in any exposed organism.

## 1. Introduction

Bisphenol A (BPA) is an endocrine disruptor (EDC), whose use is authorized in materials entering into contact with food in the European Union (EU), in accordance with Regulation (EU) no. 10/2011 [1] on material and plastic objects destined for that purpose. In January 2011, the European Commission banned the use of BPA in the manufacture of polycarbonate feeding bottles for infants. In February 2018, the EU introduced stricter limits for BPA in materials associated with food conservation, derived from the temporary tolerable daily intake (TDI) established by the European Food Safety Agency (EFSA) in 2015. Since September 2018, BPA has been prohibited in bottles and plastic containers of food for infants, and for children under 3 years of age [2]. Its use was also constrained in thermal paper as from January 2020, in the European Union [3]. Regarding toys manufactured with BPA-based polymers, and given that the final users comprise a population group at great risk, in the EU, there is currently a limit on the amount of BPA that can become detached from toys for children of up to three, which has been fixed at 0.04 mg/L since 26 November 2018. This order was adopted in accordance with Directive 2017/898 of 24 May 2017 [4]. The two main uses of BPA, accounting for approximately 95% of its production, are in the manufacture of polycarbonate plastic and of epoxy resins [5]. About 70% of the BPA produced in the industry is used to make the former, and approximately 25% is found in the latter. The remaining 5% is found in a wide variety of products, including phenolic resins, unsaturated polyester resins, can coatings, as an antioxidant and end-polymerization inhibitor in the manufacture of polyvinyl (vinyl chloride) plastics, as an intermediate in the manufacture of thermal paper, and in car tires or flame retardants [6]. The emission of BPA into the atmosphere occurs mainly because of industrial activity. In a study in which the atmospheric levels of BPA in samples collected on five continents were evaluated, pg/m^3^ was detected in coastal areas, the highest levels being reported on the east coast of Asia. The areas of large agglomerations in Asia, New Zealand, and the United States presented levels of 170–880 pg/m^3^, detecting mean concentrations of 4.55 ng/m^3^ in urban areas of India [7]. Regarding water and effluents, BPA usually appears in surface waters in low concentrations. The occurrence of various EDCs, including BPA, was studied in 14 rivers in Portugal. Analyses of the samples revealed widespread contamination by BPA, with the highest concentration reaching 98.4 ng/L. Investigations conducted in 16 large rivers in Taiwan determined BPA concentrations ranging from 0.01 to 44.65 g/dm^3^, whereas, in the sediments, they were 0.37–491.54 g/kg. In a similar context, in an analysis of surface, river, and spring water in Poland, BPA was found in all the samples tested at concentrations ranging from 6–427 ng/L in surface water to 629 ng/L in stream water [8,9].

As a consequence of the enormous production of BPA, and the multiple uses of this compound, it is found everywhere in the environment and in food, with both the human and the animal populations being continuously and inadvertently exposed to this endocrine disruptor. Due to its significant effects on health, attempts have been made to find alternatives to its use, although there is currently no safe option for this compound, despite a large number of studies focusing on the search for safe exposure levels [10,11,12]. BPA is considered to be an “emerging pollutant” to which we are constantly being exposed, and the environment, animals, and people can be affected by its possible harmful effects [13,14]. The effects on reproduction of BPA, at which most of the studies have initially been aimed, are well known. However, for some years, research has gone deeper into the effects of BPA at other levels, since it has been possible to verify how certain levels of exposure would not produce harmful effects on the reproductive system, whereas they could be observed at other levels, such as on the immune system [6,15,16].

It is, therefore, very important to find out the effects of BPA on health at different levels and systems, thus avoiding their underestimation at certain levels of exposure. In this sense, the EFSA experts, in their latest 2023 report, concluded that people of all age groups with medium and high exposure to BPA would exceed the new TDI, which is a reason for concern in terms of health.

In investigating the effects of BPA, the “One Health” perspective acquires special relevance. Studying them on different biomodels, as well as monitoring the exposure of certain animal species, could serve to understand their possible impact on the human population, in addition to evaluating the likely incidence of inadvertent exposure to BPA in the conservation of biodiversity. The objective of this work was to carry out a review highlighting appropriate studies on the relationships between BPA and its important adverse effects on health. For this purpose, we took studies published during the last 10 years, in which a wide range of doses was used in different animal models and human monitoring studies, from different bibliographic databases (Scopus, Pubmed, WoS and Science Direct). In addition, we consulted EFSA reports and pages from the Official Journal of the European Union, as well as official legislation websites.

## 2. Health Effects of Bisphenol A

**Figure 1:**
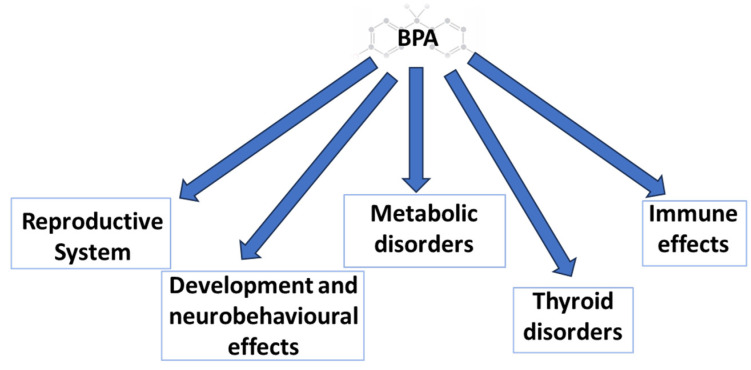
BPA multisystem toxicity.

Exposure to BPA through different routes has demonstrated its impact on both human and animal health, describing effects on different systems and organic levels. Prominent effects described are (1) those on reproduction, (2) those on development, (3) transgenerational and multigenerational effects (4) those on the metabolism, (5) immunological effects, (6) those on the thyroid function, and (7) those on oxidative stress and inflammation, among others (Figure 1) [11,17,18].

### 2.1. Effects on the Reproductive System

**Figure 2:**
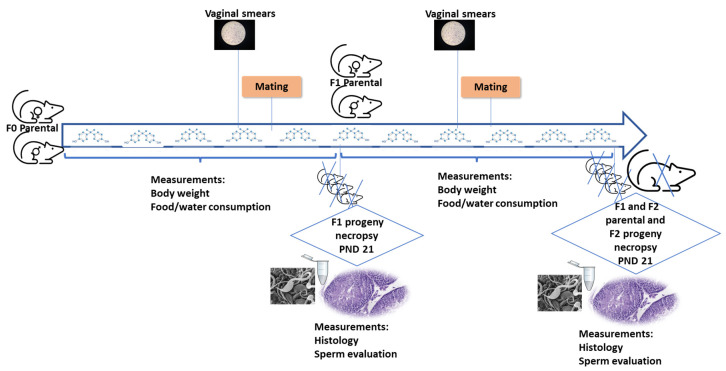
Diagram of a study on reproduction toxicity in two generations (see OCDE 416 “Two-Generation Reproduction Toxicity” [29]).

Many studies have shown that the reproductive system is affected by BPA [19,20,21,22,23]. Figure 2 depicts a reproduction toxicity study in two generations. In studies on humans, the most outstanding effects are its disruption of sex hormone activity, and its influence on the development and function of the reproductive system. In this context, it has been proposed that BPA could increase serum estradiol (E2), progesterone, luteinizing hormone (LH), and testosterone (T) levels, as well as decrease cortisol concentrations [20]. Other studies, however, revealed that BPA would reduce serum T concentration and increase E2 concentration [24].

Furthermore, it is important to mention that estrogens are largely responsible for the development of the sexually dimorphic anatomical, functional, and behavioral characteristics that are essential for reproduction in vertebrates. For instance, a key enzyme in estrogen synthesis is cytochrome P450 aromatase [25]. Aromatase converts androstenedione to estrone (E1) or testosterone (T) to 17β-estradiol (E2), the major estrogens in mammals. Aromatase activity is found in invertebrate brain and gonadal tissue; however, in mammals, including humans, this enzyme is also active in the placenta, adipose tissue, and fetal liver [25]. In healthy human breast tissue, aromatase expression is regulated by promoter regions via the protein kinase A (PKA) phosphorylation pathway. In addition, the bioactive lipid prostaglandin E2 (PGE2) has been shown to regulate the activity of the aromatase enzyme [26]. PGE2 is generated by the activity of cyclooxygenase-2 (COX-2), the rate-limiting enzyme that catalyzes the conversion of arachidonic acid to proteinoids [27]. COX-2 inhibition negatively regulates aromatase activity and decreases tumor Leydig cell proliferation [28], which suggests that COX-2 would play an important role in aromatase synthesis and steroidogenesis.

In animal experiments, exposure to BPA has been shown to have adverse effects on the reproductive system [10]. In female reproduction, neonatal or perinatal exposure to BPA has been reported as causing significant histological changes in the reproductive tract [30,31], alteration in the cyclicity of heat [32], decreased reproductive capacity [33], and changes in hormone levels in adult life [34]. In the ovary, interruption of follicular development and reduction in the number of primordial follicles [35,36] have been noted. In the case of males, an increase in the weight of the prostate, a decrease in the weight of the epididymis, a reduction in the production of spermatozoa, and a decrease in the concentrations of LH and testosterone in the blood serum have been found [37,38].

#### 2.1.1. Effects on the Ovary

Experimental animal studies indicate that prenatal and postnatal exposure to BPA would cause decreased ovarian weight, follicle number, and primordial follicle recruitment, as well as an increased corpus luteum number, even at low doses [39,40,41]. In different mammalian species, BPA decreases the number of primordial follicles and induces atresia; in both mice and lambs, this effect has been related to the acceleration of follicular recruitment. In addition, exposure to very high doses of BPA (300 mg/kg) in rats would cause an increase in cystic follicles and a decrease in corpora lutea and antral follicles [42]. In addition, BPA has been seen to reduce the number of primary and secondary follicles, as well as increase DNA damage biomarkers, suggesting that this could be an ovarian toxicity effect [43]; in fact, some studies affirm that these impacts on the ovary could be transgenerational. Thus, a study on BPA exposure in the ovaries of F1 generation mice found effects on the ovary in the F3 generation [30]. Numerous studies have concluded that BPA affects follicular growth and increases the number of atretic follicles [23,35,44,45].

Another of the associations most studied is the correlation between the highest levels of BPA in serum with the appearance of the polycystic ovarian syndrome (PCOS). PCOS is a highly prevalent disorder that affects women of a reproductive age, and it is clinically characterized by hyperandrogenism, ovulatory dysfunction, and polycystic ovary morphology, together with dysfunctions related to metabolism such as hyperinsulinemia, obesity, and insulin resistance. BPA mimics the activity of 17-βestradiol, and it has been observed to have the ability to interrupt steroid feedback at the hypothalamic–pituitary level and steroid action at the ovarian level, which would suppress the pituitary–ovarian axis functions [46]. This includes hypersecretion of circulating LH and increased follicle-stimulating hormone (FSH) levels, which would cause altered LH:FSH ratios [47]. In addition, BPA contributes to the metabolomic profile seen in PCOS, which includes insulin resistance, association with obesity, and chronic inflammation. BPA can affect ovarian steroidogenesis in the case of there being exposure during the periods known as “critical exposure windows”, which would lead to irreversible effects, as demonstrated by Fernández et al. [17], who investigated the effects of neonatal exposure to BPA in female Sprague-Dawley rats, which exhibited a PCOS-like syndrome during adulthood. This suggests a possible relationship between the development of a PCOS-like syndrome and early exposure to BPA.

In relation to oocytes, some studies have demonstrated that BPA alters their meiotic division [48]. Along the same lines, Nakano et al. [49] analyzed the effects of exposure to BPA on mice oocyte maturation in vitro. The oocytes were cultured in the presence of BPA (2, 20, 50, or 100 µg/mL) for 18 h. At concentrations of 50 and 100 µg/mL, BPA inhibited oocyte maturation and caused cell-cycle delay under these conditions. In animal studies, BPA has also been known to disrupt the transition from prophase I to MII in oocytes, with this adverse effect being related primarily to altered microtubule organization [48,49,50,51].

#### 2.1.2. Effects on the Uterus

A large number of experimental animal studies have shown that exposure to BPA produces adverse effects on the uterus. Neff et al. [52] demonstrated that exposure to environmentally relevant doses of BPA (60 µg/kg/day) had an uterotrophic effect in mice that showed increased proliferation in the uterine glandular epithelium, which is the site of origin of endometrial hyperplasia and cancer. This finding has been backed up by studies using low- and high-dose exposure in rodents (0.004 and 40 mg/kg/day) [53]. In studies conducted in CD-1 mice, exposure to low doses (60–600 µg/kg/day) of BPA increased glandular density, peri glandular collagen accumulation, and abnormal functions of the endometrial epithelium and stroma [54], indicating that BPA could have an inducing role in endometrial cancer [55].

#### 2.1.3. Effects on the Placenta

BPA exposure has been linked to certain obstetric complications associated with the placenta, including preeclampsia, decreased fetal growth, miscarriage, and preterm birth. The current findings suggest that BPA would cause pathological changes in the placenta by interrupting its metabolic activities. Depending on the exposure concentrations, BPA could trigger apoptotic or antiapoptotic signals in trophoblasts, potentially interfering with pregnancy [56].

In the last decade, several studies have examined the effects of BPA on mouse placenta [57,58,59,60,61]. Many investigations showed that BPA interferes with the placental epigenome of mammals. Strakovsky et al. [62] found that BPA affected placental imprinting loss and decreased DNA methylation. In the placental IGF2/H19 domain, loss of imprinting and decreased methylation led to disrupted nutrient allocation and impaired fetal growth. Likewise, in that study, in placentas of the same age, without fetal malformations, they found that there were miR-146a alterations after exposure to BPA.

Other female reproductive abnormalities due to perinatal BPA exposure include early-onset vaginal opening and puberty, as well as alterations in estrous cyclicity, plasma LH levels, vaginal and uterine histology, and mammary gland, uterus, and ovarian morphology [63,64].

#### 2.1.4. Effects on the Fetus

##### Birth Weight

Multiple studies in women have shown a negative correlation between BPA concentrations in amniotic fluid and urine and birth weight [65,66]. On the contrary, in some works, no association has been reported between BPA concentrations in maternal serum and urine at the beginning of gestation and at birth, with a lower weight than expected for gestational age [67,68].

Hu et al. [69] observed a significant dose–response curve for reduced birth weight after BPA exposure during gestation. In this study, a total of 452 mother–child pairs were selected in the city of Wuhan, China during 2012–2014. Mothers with low-birth-weight infants had significantly higher urinary BPA levels (4.70 µg/L) compared to mothers in the control group (2.25 µg/L). That association was more pronounced among female infants than among male ones, evidencing the relationship between the highest levels of BPA in urine and the sex of the offspring with a lower weight. Scientific research in this regard has been focused on determining the concentration of BPA in the placenta. In their study, Troisi et al. [70] analyzed placenta samples from 200 individuals, with BPA levels being measured by gas chromatography/mass spectrometry (GC–MS). Additional data on the mother and infant were collected from medical records and correlated with BPA levels in the placenta. The results of this study gave a significant negative correlation between the calculated birth weight percentile and placental BPA levels. Low-birth-weight infants and those small for their gestational age also had significantly higher placental BPA concentrations compared with infants of a normal weight or average/large for their gestational age.

In animal studies, it has been found that early exposure to BPA probably influences several important mechanisms for body weight regulation, including adipocyte deposition, glucose uptake, and homeostasis. Susiarjo et al. [71] demonstrated that BPA exposure during gestation and lactation affected postnatal growth of offspring of C57BL/6 mice. In that study, F1 males exposed to the lowest doses (10 µg/kg/day) of BPA exhibited accelerated weight gain after weaning, with no difference observed in food intake of F1 male mice in the different exposure groups. This effect appears to be sex-dependent, as no significant differences in body weight, body fat content, or bone mineral content and density were detected in females.

##### Premature Labor

There are few data on the correlation between preterm birth and BPA levels in pregnant mothers [72,73,74]. However, Smarr et al. [74] showed that this weak correlation is stronger for newborn girls than for boys.

In a similar context Behnia et al. [75] evaluated plasma and amniotic fluid in a sample of pregnant mothers, showing that mothers with higher plasma concentrations of BPA had a risk of a shorter gestation or premature rupture of membranes. Furthermore, some studies such as that of Cantonwine et al. [72] and Weinberger et al. [73] described an inverse correlation between the concentration of BPA in maternal urine and the length of gestation. However, the results of some research with animals indicate that, in females exposed to high concentrations of BPA, there was a lengthening of the gestation period, although this fact could not be directly attributed to exposure to BPA [11].

##### Fetal Malformation

Several studies have observed BPA-related fetal malformations, which would suggest that this endocrine disruptor is transferred trans-placentally to the embryo–fetal compartment [76]. Guida et al. [77] investigated total, free, and conjugated BPA measured in the blood of 151 pregnant women divided into two groups: one with an established diagnosis of the developmental defect, and the other with the normally developing fetus. The results showed that free BPA was higher in the blood of women pregnant with a fetus with chromosomal malformations and those of the central and peripheral nervous system, compared to women in the control group. This suggests a greater susceptibility to abnormalities among “poor metabolizers”, which could be due to the free fraction being the active one, i.e., the one capable of binding to its sites of action and producing its adverse effects. Having a higher free proportion implies that a larger amount of BPA is able to reach its action sites. In addition, BPA possibly interferes with the progression of meiotic maturation (as demonstrated in vitro) and causes alterations in spindle organization and chromosome alignment.

On another note, the effect of BPA on male genital malformations has been demonstrated in rats and humans [78,79]. In human male studies, Fernández et al. [80] observed an increased risk of genital malformations due to high placental concentrations of BPA. Furthermore, Mammadov et al. [81] correlated decreased anogenital distance (AGD) in male offspring with high parental exposure to BPA. Cryptorchidism and hypospadias are among the most frequent neonatal malformations associated with multiple exogenous factors although they could not be significantly correlated with BPA exposure. Dobrzyńska et al. [82] demonstrated, in a study with mice, the parents (males) of which were exposed for 8 weeks to 5, 10, and 20 mg/kg bw of BPA, that the frequency of abnormal skeletons in the F1 offspring increased dose-dependently, observing malformations such as the concavity of the parietal bones and the presence of extra ribs.

#### 2.1.5. Effects on Male Reproduction

Various in vivo and in vitro studies suggest that BPA and its analogs have deleterious effects on the male reproductive function and sperm quality. In this sense, it is known that BPA antagonizes endogenous hormones and interferes with steroid-mediated processes that affect male reproduction. Chianese et al. [83] demonstrated the existence of alterations in the testes in male rats after exposure to BPA from the fetal period to sexual maturation, demonstrating an alteration in the cytoarchitecture of the seminiferous epithelium. The oxidative stress mechanisms and a massive production of reactive oxygen species (ROS) would explain the interruption of functional communications between Sertoli and germ cells, as well as the alteration of spermatogenesis and cell damage in post-meiotic stages. Zhang et al. [84] demonstrated that BPA would increase the number of germ cells entering meiosis, causing an abnormal state of proliferation. The production of ROS in the testes and the DNA damage in post meiotic spermatids would justify the fact that BPA can induce the formation of poor-quality sperm, with possible transgenerational effects on the offspring.

Regarding Leydig cells, the main effects of BPA exposure on their activity are the alteration of the hormonal microenvironment in the testes, and the upregulation of key steroidogenic enzymes. In addition to Sertoli and germ cells, Leydig cells are also targets for BPA. The main effects of the latter’s exposure on Leydig cell activity are the alteration of the hormonal microenvironment in the testes and the upregulation of steroidogenic enzymes, with an increase in estrogen production. In this sense, Lan et al. [85] demonstrated that the ratio of sex hormones (testosterone/estradiol) decreased in male rats administered for 5 days with BPA doses of 0.5 µg/kg/day. In their study, they observed that the ratio of sex hormones (T/E) was dramatically reduced in the BPA group compared to the control.

In humans, BPA has been known to reduce sperm quality, decrease sexual function, and reduce fertility. The correlation between BPA exposure and decreased semen quality, assessed as sperm count, motility, and vitality, has been seen to impact capacity and acrosomal reaction [86]. In particular, BPA modulates the motility of human spermatozoa in vitro, affecting their mitochondrial potential in a pathway that involves free Ca^2+^ as a second messenger [87]. Ji et al. [88] conducted a cross-sectional study in the Chinese city of Sandu with a sample of 774 men between the ages of 18 and 55, who underwent a semen analysis, and their BPA levels were associated with linearity, oscillation, amplitude of lateral displacement of head, mean angular displacement, and sperm concentration. This is consistent with many other studies showing that infertile men had higher levels of BPA in urine and plasma [89]. In general, BPA could affect the normal reproductive function by altering the activity of sex hormones, even triggering the onset of infertility. Similarly, Manfo et al. [90] found that men exposed to BPA had reduced libido levels, increased erection and ejaculation difficulties, and decreased satisfaction with their sex life.

### 2.2. Developmental and Neurobehavioral Effects

Maternal exposure to BPA and its level in umbilical cord blood have been reported to have a sex-specific effect on shortened AGD in children [91]. Barrett et al. [92] found that girls who had been exposed to higher levels of BPA during the first trimester of pregnancy had a shorter AGD. In rodents, Liu et al. [93] demonstrated that, in knockout mice exposed to BPA at a dose of 100 mg/L/day per gavage, the anogenital distance was shortened, and both the male testicular weights and the testosterone levels were reduced. The development of puberty has also been linked to the levels of exposure to BPA. Chen et al. [94] found that BPA levels were associated with idiopathic central precocious puberty (PPCI) in school-age girls. However, the study by Berger et al. [95], found that higher BPA concentrations could induce delayed puberty in girls and earlier puberty in boys, demonstrating a sex-dependent effect.

In addition, the neuroendocrine regulation inhibition induced by BPA could cause a series of mental and behavioral alterations in offspring. Chen et al. [94] investigated the effects of gestational exposure to BPA related to mental and behavioral problems in preschool children. They found that increased maternal exposure to BPA could be a potential risk factor for unusual conduct in children, especially boys, of preschool age. Perera et al. [96] found that prenatal BPA exposure was significantly associated with depression and anxiety in children, whereas postnatal BPA exposure was not related to these illnesses. Therefore, exposure to BPA during gestation, the critical exposure window, could interfere with brain development in offspring and increase the risk of behavioral problems. In addition, it was suggested that BPA could be associated with hyperactivity disorder, antisocial behavior, problems related to sleep, and language development [94,97,98].

Animal studies have reported that exposure to BPA during the prenatal, postnatal, and juvenile periods causes neurotoxic effects on the brain, and behavioral changes. Zhang et al. [99] in a multigenerational study with mice observed that even a low dose of maternal exposure to BPA (0.5 µg/kg/day) could significantly affect, depending on sex, the learning and memory capacity of male F1 mice, but not of the F2 generation. They also observed a decrease in the number of neurons in the hippocampus of the F1 and F2 generations after maternal exposure to BPA, and DNA damage to brain cells, but only in the F1 offspring. In addition, according to these authors, maternal exposure to BPA could lead to variations in hippocampal neurotransmitter levels, indicated by a decrease in the glutamic acid/gamma aminobutyric acid (Glu/GABA) ratio in F1 offspring. Bi et al. [100] exposed transgenic mice orally to BPA (0.05 mg/kg/day) from postnatal day (PND) 0 to PND 60, subsequently subjecting them to behavioral tests. Their results suggest potentially detrimental effects after BPA exposure on the excitatory neuronal circuitry in spatial memory formation.

Neurobehavioral and adult brain effects are mostly due to BPA exposure during critical life stages; they are subsequently transmitted to their offspring and can persist or further develop in adulthood. In this regard, Wang et al. [101] demonstrated that intrauterine exposure to BPA in adult CD-1 mice induced permanent changes in the gene expression in the brain, including a significant diminution in motor activity, in learning capacities, in long-term memory, and an increase in anxiety in young mice evaluated at 18 months of age. Fetal exposure, and that in the first years of life, permanently affected neurobehavioral functions, which deteriorated with age. They, therefore, revealed that BPA exposure could worsen aging effects, long-term memory, and an increase in anxiety.

In addition, it has been demonstrated that, after exposure during adult life, similar effects are produced. For example, Ni et al. [102] exposed adult, male and female, C57BL/6J mice at 8 weeks of age to 0.05, 0.5, 5, and 50 mg/kg of BPA during 22 weeks, and observed that exposure impaired the memory and learning capacity of the male mice, which was associated with an increase in neuroinflammation and a harmed hemato-encephalic barrier.

In studies with fish, authors such as Heredia-García et al. [103] evaluated the neurotoxic effects of acute exposure to BPA (96 h) at environmentally relevant concentrations (220, 1180, and 1500 ng/L) in adult zebrafish (*Danio rerio*), subsequently performing swimming behavior assessment tests (novel tank). Their results indicated that exposure to 1500 ng/L of BPA reduces the total distance traveled and increases the stopping time of the fish, concluding that environmentally relevant BPA concentrations could cause anxiety-like behavior and neurotoxic effects in adult zebrafish.

On the same lines of the study on the effects on the brain in the adult animal, Schirmer et al. [104] studied the effects, in the goldfish (*Carassius auratus*), after 1 month’s exposure to environmentally relevant concentrations of BPA (1 and 10 µg·L^−1^) on the Mauthner neurone, an essential one in vertebrates to trigger “flight from predators” behavior. Their findings demonstrated that this exposure for 1 month strongly affected visual and acoustic processes occurring in those neurons, thus generating an impact on the essential communication functions in the brain of adult vertebrates. These effects, nevertheless, were not produced after acute exposure (1 h) in those same vertebrates.

### 2.3. Transgenerational and Multigenerational Effects

The multigenerational and transgenerational inheritance mechanisms of abnormal developmental phenotypes include epigenetic misregulation in germ cells. Multigenerational effects involve direct exposure of the factor influencing in the development of the disease, in contrast to transgenerational effects, in which transmission between generations does not imply direct exposure. Very many studies support the theory that BPA could alter epigenetic marks in rodents and humans. These epigenetic marks include DNA methylation, histone post-translational modifications, and noncoding RNAs [105,106]. The transgenerational effects of BPA have been demonstrated in mammals and nonmammals [107,108]. There is also evidence of multigenerational and transgenerational inheritance of abnormal developmental changes in offspring after exposure to this endocrine disruptor. BPA exposure is related to the transgenerational inheritance of reproductive, metabolic, or neurological phenotypes [22,63,109,110].

In rodents, developmental BPA exposure has been associated with social recognition and behavioral differences in three-generation studies [107]. In that study, after exposing the mothers to concentrations of 20 µg/day during mating and gestation, it was observed that, in subsequent generations of animals, juvenile mice exposed to BPA showed more active exploratory behavior than controls, without finding any sex differences in the performance of any of the behavioral tests carried out. These results demonstrated that exposure to BPA during pregnancy has lasting transgenerational effects on social recognition and activity in mice.

**Figure 3:**
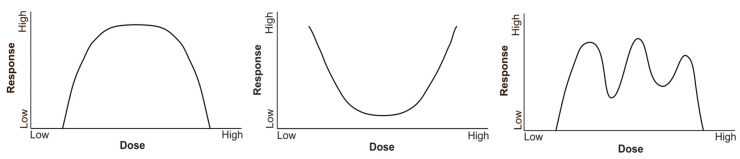
Example of nonmonotonic behavior dose–response curves (see [11,113,114]).

Various studies have shown that intrauterine exposure to BPA affects the reproductive function in developing mice, and that function can be altered for up to three generations [111,112]. Mahalingam et al. [111] exposed FVB mice to BPA at low concentrations (0.5, 20, and 50 µg/kg/day orally) to investigate the multigenerational effects of BPA on the F1, F2, and F3 generations. The results revealed that intrauterine BPA exposure decreased cytochrome P450 aromatase and estradiol mRNA levels in the F1 generation. Likewise, it was found that this exposure decreased testosterone levels and altered the mRNA levels of several steroidogenic factors in the F2 generation, producing multigenerational effects on the ovary and steroidogenesis in mice up to the F2 generation. In adulthood, effects were observed on the reproduction indices in generations F2 and F3, such as a decrease in fertility. Of note is that some of the most pronounced effects were measured in mice whose parents received the lowest dose of BPA. This would be related to the results that back up the theory that BPA shows a nonmonotonic dose–response curve. A monotonic response is characterized by a slope that does not change its sign. In contrast, a nonmonotonic dose–response curve (NMDRC) is characterized by a slope that changes its sign within the tested dose range. Some curves are U-shaped, some are inverted U-shaped, and, in others, the sign of the curve may change at multiple points throughout the range of doses studied. One of the characteristics of endocrine disruptors, specifically BPA, is that they produce nonmonotonic dose–response curves (Figure 3).

Furthermore, Shi et al. [115] examined the transgenerational effects of BPA on male reproductive functions using CD-1 mice as a model, and exposing them orally to BPA and other analogs, at concentrations of 0.5 and 50 µg/kg/day and using mice of F1 and F2 offspring to generate F3 males. Prenatal exposure to BPA was found to decrease sperm count and motility, and interrupt the progression of germ cell development in F3 males. Deregulated serum levels of 17β-estradiol and testosterone were also observed, as well as steroidogenic enzyme expression in adult testes in F3. The results of this study suggest that prenatal exposure to BPA would originate transgenerational effects on male reproductive functions due to an altered epigenetic modification in neonatal and adult testes.

Rahman et al. [112] investigated the histological changes in the testes, together with the functional, biochemical, and epigenetic (DNA methylation) properties of spermatozoa from male CD-1 mice exposed to BPA (4 and 50 mg/kg/day) for 6 weeks and crossed with untreated females to produce up to a third generation (F3). The results showed that paternal exposure to BPA disrupted spermatogenesis, leading to a decrease in the total sperm count of the F0–F2 offspring. In addition, they demonstrated that a dose of 50 mg/kg/day decreased sperm motility in F0–F2 males by mediating the overproduction of ROS. BPA was also shown to compromise sperm fertility in F0–F2 generation mice in both dose groups, but in F3 only in the high-dose group.

In this context, Bansal et al. [110] used C57BL/6J (F0) mice that were exposed to BPA doses of 10 µg/kg/day (LowerB) and 10 mg/kg/day (UpperB), to study the effects on the pancreatic islets of offspring in the first (F1), second (F2), and third generations (F3). Male F1 and F2 offspring exposed to the low dose of BPA had reduced β-cell mass and smaller islets, which was associated with increased glucose-stimulated insulin secretion. The same did not occur in the case of females, which exhibited comparable results with controls.

The transgenerational effects of BPA in relation to neurobehavioral actions have also been described in many studies. In this line, Wolstenholme et al. [116] examined social recognition behaviors in third-generation mice after gestational exposure to BPA. In their study, they demonstrated that transgenerational exposure to BPA interrupted social interactions in mice and deregulated the normal expression of genes involved in excitatory postsynaptic density (PSD), closely related to neurobehavioral disorders during neuronal development.

### 2.4. Metabolic Effects

Experimental studies have shown that exposure to BPA generates weight gain, changes in blood glucose levels, and insulin resistance, as well as the development of dyslipidemia and alterations in lipid metabolism [117,118,119]. Epidemiological evidence found positive associations between BPA exposure and the onset of metabolic diseases such as obesity and type 2 diabetes. Likewise, numerous animal studies indicated that BPA exposure resulted in glucose intolerance, insulin resistance, and modifications in glucose homeostasis [120,121]. The alteration of different biochemical parameters could likewise be observed throughout different generations and be considered an indirect indicator of effects derived from chronic exposure to BPA. In this respect, Bujalance et al. [11], in a study of several generations of mice exposed to different concentrations of BPA (0.5, 2, 4, 50 and 100 µg/kg/day), reported that alterations were produced in the biochemical parameters throughout the generations exposed, modifying glucose, albumin, and total protein levels. Regarding glucose levels, it could be said that exposure to BPA causes a hyperglycemic effect, possibly due to an alteration in glucose metabolism in the pancreas. In addition, the variations in the levels of total proteins and albumin could be explained by the action of BPA, which would give rise to an alteration in the liver, resulting in a modification mainly in protein synthesis.

Exposure to BPA produces obesogenic effects, which not only occur in exposed subjects, but could also cause transgenerational outcomes. A direct interruption of endocrine regulation, of neuroimmune and signaling pathways, as well as of the intestinal microbiota, has been identified as being due to exposure to BPA, which would lead to overweight or obesity creating, in these cases, cardiovascular complications, one of the main consequences derived from obesity [122].

As a consequence of these obesogenic effects, there is an alteration in energy homeostasis, the lipid composition of the liver, and insulin signaling in insulin-sensitive organs such as the liver, muscle, and adipose tissues [123]. Furthermore, the link between energy homeostasis and reproduction has been demonstrated, in both animal and human models, with leptin, a peptide hormone produced in white adipose tissue, which is the main peripheral biomarker of metabolic status. Leptin (adipokine involved in the control of food intake through appetite suppression) can also stimulate oxidative stress, inflammation, thrombosis, arterial stiffness, angiogenesis, and atherogenesis. These leptin-induced effects could lead to a predisposition to suffer from cardiovascular diseases. Leptin levels have been positively associated with the presence, severity, extent, and complexity of coronary atherosclerosis lesion, as well as the presence and severity of ischemic and hemorrhagic strokes. In addition, leptin has been shown to independently predict common carotid intima–media thickness and carotid plaque instability. Elevated leptin levels have also been linked to the incidence and progression of chronic kidney disease, as well as insulin resistance, type 2 diabetes, and microvascular and macrovascular diabetic complications.

BPA directly affects food intake by modulating the activity of metabolic sensors produced in the arcuate hypothalamic nucleus (ARC), thus interfering in the interaction between the gonadotropin-releasing hormone (GnRH) and neural networks involved in the metabolic control of reproduction [124,125].

In studies on humans, BPA has been confirmed as being closely related to some metabolic diseases by altering the neuroendocrine function, potentially representing an important factor leading to the development of chronic diseases. Aktag et al. [126] found that urinary BPA levels in children during their prepubertal stage were positively associated with metabolic syndrome (MetS). Shu et al. [127], for their part, demonstrated that the serum concentration of BPA was positively associated with the increase in fasting plasma glucose levels. This study suggested that BPA might contribute to obesity and the development of type 2 diabetes with insulin resistance.

In animal studies, Moon et al. [120] demonstrated that oral exposure to BPA in mice produced glucose intolerance and insulin resistance. Yang et al. [128], observed that BPA promoted adiposity in 5 week old mice exposed to doses of 5–5000 µg/kg/day of BPA with a low-calorie diet in a nonmonotonic dose–response manner. Shu et al. [129] demonstrated that prenatal exposure in mice to BPA doses of 5 mg/kg/day by oral gavage caused transcriptomic and methylomic alterations in the liver, adipose tissue, and hypothalamus of the male offspring, with inter-tissue alterations in the metabolism of the lipids, and tissue-specific alterations in glucose metabolism, histone proteins, and the extracellular matrix.

Regarding the different metabolic alterations associated with exposure to BPA, high concentrations of this endocrine disruptor in urine were correlated with higher levels of blood pressure, indicating that it could possibly contribute to the development of cardiovascular diseases [130]. Therefore, the peripheral arterial disease (PAD) would be positively associated with urinary BPA levels, which could be used as a subclinical measure of atherosclerotic vascular disease, since there are positive associations, demonstrated in epidemiological studies, between urinary or blood levels of BPA and the development of coronary artery stenosis, carotid atherosclerosis, and peripheral kidney disease. Recent epidemiological studies have also verified that urine or serum BPA levels were positively associated with coronary artery stenosis, carotid atherosclerosis, and peripheral artery disease, suggesting that BPA exposure might be an emerging risk factor for the development of atherosclerosis [131].

Lastly, it should be noted that bone metabolism could also be affected by BPA through the interruption of calcium phosphate metabolism and the consequent reduction in bone mineral density [89]. It is known that BPA interferes with bone modeling and remodeling by altering hormonal regulation and causing the epigenetic alteration of target genes, which would cause osteoporotic lesions [132]. In particular, BPA has been shown to exert an estrogen-like action via estrogen receptors α and β (ERα and ERβ) and ER-related γ (ERRγ); however, unlike estrogens, it harms concomitant bone development with the inhibition of osteogenic gene expression levels [133,134], which would indicate that, after exposure to BPA, the agonist effects induced on the ER could inhibit osteogenesis, increasing bone loss.

### 2.5. Immunological Effects on Oxidative Stress and Inflammation

BPA is closely linked to immune function, inflammation, and oxidative stress [15,135]. It can also induce mitochondrial damage and cell apoptosis [136]. Xu et al. [137] found that immune cell populations and innate and adaptive immune system functions were altered upon exposure to BPA during development, including decreased regulatory T cells and upregulated proinflammatory and anti-inflammatory cytokines and chemokines. In 2019, this same research group found that BPA modulated immune function and the gut microbiome, which would be closely associated with a higher incidence of type 1 diabetes. In their study on animals, they demonstrated that BPA exposure caused a change in proinflammatory factors in females, while, in men, the same exposure caused elevated levels of anti-inflammatory immune factors and a decrease in the proinflammatory gut microbiome [138].

In this respect, taking into account the evidence from animal data and observational studies in humans, the immune system has been identified as being the one most sensitive to BPA exposure. One effect on T helper 17 lymphocytes (Th17 cells) in mice was identified as being critical; these cells are vital in cellular immune mechanisms and are involved in the development of inflammatory conditions, including autoimmunity and lung inflammation. On the basis of all this scientific evidence, the EFSA experts have established a TDI of 0.2 ng/kg/day, replacing the previous temporary level of 4 µg/kg/day, establishing in April 2023 a TDI approximately 20,000 times lower than that previously established [6].

Oxidative stress is another BPA toxicity mechanism. As a metabolic and endocrine disruptor, BPA alters redox homeostasis by increasing oxidative mediators and reducing antioxidant enzymes, leading to mitochondrial dysfunction, disruption of cell signaling pathways, and induction of apoptosis. Antioxidant enzymes are a class of essential enzymes that can protect against oxidative damage. BPA could reduce these antioxidant enzymes and increase free-radical generation and lipid peroxidation, leading to oxidative stress damage [135,139]. BPA can disrupt the balance of the antioxidant system and cause adverse effects by inhibiting antioxidant enzyme activity, downregulating antioxidant gene expression, and lipid peroxidation (LPO) formation.

Kaur et al. [140] observed that BPA could be an environmental risk factor for autism by inducing oxidative stress and mitochondrial dysfunction. Kazemi et al. [141] showed that BPA could promote the generation of ROS and increase the expression levels of the antioxidant gene in liver tissue, causing hepatotoxicity. In the study by Yuan et al. [142], BPA was found to cause oxidative stress in the testis and to reduce hydrogen peroxide (H_2_O_2_) primarily through the stimulation of catalase (CAT) activity. In animal models, it has been observed that chronic exposure to BPA could increase the levels of malondialdehyde (MDA) and IL-18, as well as reduce the levels of superoxide dismutase (SOD) in the lung tissue of adult male rats, which could result in inflammatory lung diseases [143]. BPA may also increase glutathione (GSH) content, CAT activity, and the formation of mitochondrial reactive oxygen species (ROS) and LPO in the kidney of adult male Wistar rats, leading to impaired renal function [144].

### 2.6. Effects on Thyroid Function

The thyroid hormone is essential for development, growth, and metabolism, and it plays an especially important role in neurodevelopment. Therefore, the alterations that may exist in the function of the thyroid hormone could hamper these vital functions. BPA can interfere with thyroid function through several mechanisms. It can inhibit thyroid hormone synthesis by altering thyroid hormone regulation through its interference at the pituitary and hypothalamic levels [145]. Less likely, it can also interfere with thyroid hormone transport and metabolism, although it is thought that the T_3_ nuclear receptor (TR) antagonist effect of BPA may be the primary mechanism through which it disrupts the thyroid function.

In some animal studies, Da Silva et al. [146] administered BPA to Wistar rats, subsequently measuring their thyroid hormone levels. Exposure to BPA (40 mg/kg, 15 days, orally) in adult rats increased T_4_ levels. Furthermore, Fernández et al. [17] investigated neonatal exposure to BPA (2.5–6.2 mg/kg, 10 days, subcutaneously), observing decreased T_4_ levels and increased thyrotropin (TSH) levels in adulthood.

Likewise, maternal exposure to BPA in rats can affect thyroid hormone levels in their offspring. In a study with rats, Silva et al. [147] observed that maternal exposure to BPA during pregnancy and lactation (10 and 50 µg/kg/day, orally) decreased T_3_ and T_4_ levels in the offspring (postnatal day [PND] 15). For their part, Xu et al. [148] observed that maternal exposure to BPA induced a transient increase in T_4_ (PND7) levels, followed by a decrease in T_4_ (PND21) in the male offspring. However, other investigators showed that perinatal exposure to BPA (0.0025–40 mg/kg, orally or subcutaneously) did not alter TSH and T_4_ levels in the offspring [110,149].

In humans, studies such as the one by Sanlidag et al. [150] evaluated dose-dependent maternal exposure to BPA effects on thyroid functions in neonates. The levels of BPA, TSH, and free T_4_ were measured in the umbilical cord blood, not detecting any significant effect on thyroid hormones.

For their part, Li et al. [151], conducted an epidemiological study in China to examine the association between urinary BPA and thyroid nodules (TN) in women. A higher concentration of BPA in urine was associated with a higher risk of TN only in those with positive thyroid autoantibodies. Furthermore, this association was dose-dependent, which would indicate that any increase in BPA exposure was related to an increased risk of TN.

## 3. Conclusions

BPA is an environmental and food contaminant, which is found ubiquitously in different sources, whereby both the animal and the human populations remain continuously and inadvertently exposed to it. Numerous monitoring studies have concluded that exposure to BPA is constant; thus, the potential health effects of exposed organisms cannot be underestimated. The effects of BPA follow a nonmonotonic behavior, typical of endocrine disruptors, which do not behave dose-dependently. Among other reasons, the evaluation of the risk of exposure to this compound is more complex, and harmful effects may appear at lower doses. There are multiple effects on health at different levels and systems from this endocrine disruptor. The initial studies on it focused on evaluating its impact on reproduction, demonstrating that it interfered with fertility, and that it was related to the increase in hormone-dependent pathologies, among others. Similarly, a multitude of studies have shown that BPA exerts important effects, which could cause serious pathologies that would compromise the long-term life of exposed organisms, by triggering effects at different levels, such as metabolic, immune system, or neurodevelopmental ones, or those related to obesity, hypertension, diabetes, or diseases such as depression or anxiety, among others. The variety of pathologies with which long-term exposure to BPA is associated is so wide, acting or not at certain stages of development (critical exposure windows); hence, it is a real challenge to identify safe exposure limits, since that which may be innocuous for a certain system may not be so for another, and exposed organisms may suffer from subclinical diseases. At present, it is not known how its long-term effect will evolve in free-living animals, and research on its direct impact on the conservation of biodiversity has focused on the evaluation of toxicity in laboratory animals, or on monitoring in the human population, demonstrating its adverse effects on multiple systems. However, BPA is an “emerging contaminant”, whose action affects all exposed organisms; hence, the monitoring of these species has acquired a special relevance, making it essential to focus the studies according to the “One Health” concept. It is urgent to establish new lines of research that evaluate the effects at an environmental, animal, or human level, as well as to establish measures to prevent environmental and food contamination and foster an essential restriction of its use and the maintenance of exposure surveillance, as being conducted by regulatory agencies, while continuing to search for a safe alternative to the use of BPA.

## Data Availability

Not applicable.
